# Carriage of hypervirulent and ESBL-producing *Klebsiella pneumoniae* complex among community-dwelling individuals in Japan

**DOI:** 10.1128/aem.01687-25

**Published:** 2026-01-14

**Authors:** Akiko Watanabe, Yukio Tawada, Makoto Moriyama, Yohei Doi, Masahiro Suzuki

**Affiliations:** 1 Department of Microbiology, Fujita Health University School of Medicine 89305, Toyoake, Aichi, Japan; 2 Department of Food and Nutritional Sciences, College of Bioscience and Biotechnology, Chubu University 12740, Kasugai, Aichi, Japan; 3 Moriyama Environmental Wellness Laboratories, Nagoya, Aichi, Japan; 4 Department of Infectious Diseases, Fujita Health University School of Medicine 89305, Toyoake, Aichi, Japan; 5 Center for Innovative Antimicrobial Therapy, Division of Infectious Diseases, University of Pittsburgh School of Medicine 12317 , Pittsburgh, Pennsylvania, USA; Centers for Disease Control and Prevention, Atlanta, Georgia, USA

**Keywords:** ESBL, hypervirulent, healthy individuals, *Klebsiella pneumoniae* complex

## Abstract

Despite the increasing number of reports on hypervirulent and extended-spectrum β-lactamase (ESBL)-producing *Klebsiella pneumoniae* infections, data on the distribution of these pathogens in the community are limited. To address this knowledge gap, we investigated the carriage rates of *K. pneumoniae* complex in the stools of community-dwelling individuals in Japan. From 627 stool samples submitted to a commercial diagnostic laboratory, 407 *Klebsiella* strains were identified from 368 samples, corresponding to a colonization rate of 58.7%. Based on whole-genome sequencing, *K. pneumoniae* was the most prevalent species (*n* = 218, 53.6%), followed by *Klebsiella variicola* (*n* = 137, 33.7%). The detection rate of *K. variicola* was higher than previously reported in studies from other Asian countries. The overall distribution of sequence types (STs) was similar to those observed in previous studies of clinical isolates. However, hypervirulent *K. pneumoniae* clones, specifically ST23-K1 and ST412-K57, and ESBL-producing strains were rare, each accounting for less than 1% of the strains. These findings suggest that, while carriage of *K. pneumoniae* complex species is common in the community, healthcare settings may represent a more significant reservoir of hypervirulent and ESBL-producing *K. pneumoniae* strains in this epidemiological setting.

**IMPORTANCE:**

*Klebsiella pneumoniae* complex species are bacteria that can cause serious infections, especially in hospital settings. Some types have become more dangerous because they are resistant to antibiotics or highly virulent. To better understand where these harmful clones come from, this study looked for *Klebsiella* species in healthy people living in the community in Japan. The results showed that these bacteria are commonly found in the gut, particularly *K. pneumoniae* and *K. variicola*. While some strains with traits linked to antibiotic resistance or severe infections were identified, they were rare. These findings suggest that most people carry *Klebsiella* strains as commensals and that the more dangerous forms of *Klebsiella* are likely spreading mainly in healthcare settings.

## INTRODUCTION

*Klebsiella pneumoniae* is a well-recognized opportunistic pathogen responsible for a variety of infections, including urinary tract infections, pneumonia, and bacteremia. In recent years, species belonging to the *Klebsiella pneumoniae* complex, primarily *K. pneumoniae*, *Klebsiella variicola*, and *Klebsiella quasipneumoniae*, have received increasing attention due to their roles in antimicrobial resistance and hypervirulence. Among antimicrobial-resistant *Klebsiella*, carbapenem-resistant lineages harboring the *bla*_KPC_ gene have spread globally, with sequence type (ST) 258 identified as the predominant clone in many countries (1). In addition, the prevalence of extended-spectrum β-lactamase (ESBL)-producing strains is increasing. In Japan, approximately 18% of *Klebsiella* clinical isolates are reported to produce ESBLs (1).

Another critical concern is the emergence of hypervirulent *K. pneumoniae,* which was initially identified in East Asia and has since become a significant global public health threat due to its propensity to cause severe community-associated infections, including liver abscesses, endophthalmitis, and meningitis (2). It possesses a distinctive ability to disseminate hematogenously to multiple organs, complicating both diagnosis and treatment, and is frequently associated with high mortality rates (3). Alarmingly, recent studies have identified the emergence of multidrug-resistant (MDR) hypervirulent *K. pneumoniae* strains (4). Given the combination of enhanced virulence and limited treatment options, continued surveillance of these strains is imperative.

Hypervirulent *K. pneumoniae* strains are typically characterized by the presence of multiple virulence-associated genes, most notably *rmpA* and *rmpA2*, which regulate the expression of the mucoid phenotype. These genes contribute to the hypermucoviscous phenotype often identified by a positive string test. However, this phenotypic test alone is insufficient for definitive identification of hypervirulent *K. pneumoniae*. A more reliable definition of hypervirulent *K. pneumoniae* involves the detection of both *rmpA* and *rmpA2* genes (5). Additional key virulence factors include siderophore systems that facilitate iron acquisition, such as aerobactin (*iucABCD-iutA*) and salmochelin (*iroBCDN*). The *peg-344* gene, which encodes a putative metabolite transporter, has also been reported as a robust marker of hypervirulence (6). These virulence genes are typically encoded on the prototypical virulence plasmid pLVPK (7). Moreover, capsular genotypes K1 and K2 identified through *wzi* gene sequencing are strongly associated with the hypervirulent phenotype.

Several clonal lineages are recognized as hypervirulent *K. pneumoniae*, most notably ST23 with capsular genotype K1. K1-ST23 strains frequently harbor genes encoding multiple siderophores (e.g., aerobactin, salmochelin, yersiniabactin, and colibactin) as well as *rmpA* and *rmpA*2. Other lineages, such as ST65, ST86, and ST375, typically associated with K2 capsular genotype also exhibit hypervirulence (8, 9). These lineages have been identified mostly in East Asia, including Japan (10, 11).

The prevalence of hypervirulent *K. pneumoniae* clinical isolates varies geographically. Higher carriage rates have been reported in Asia, including 21.6–37.8% in China (12), 42% in Korea (13), and 29.6% in Taiwan (14). In contrast, lower rates are observed in other regions, such as 3.2% in Spain, 3.9% in the United States, and 4% in Iran. Several studies suggest that intestinal colonization by *K. pneumoniae* may precede subsequent systemic infection (15, 16), highlighting the need for surveillance of hypervirulent *K. pneumoniae* carriage.

In Japan, a unique system exists for monitoring intestinal carriage of pathogenic bacteria in the community: individuals working in food-related occupations are required to undergo routine stool testing, typically at least twice per year, to prevent the spread of etiologic agents of infections, such as dysentery and typhoid fever. In this study, we utilized this surveillance framework to assess the carriage rate of hypervirulent *K. pneumoniae* among community-dwelling individuals in Japan and identify potential risk of community transmission of hypervirulent and ESBL-producing *K. pneumoniae*.

## RESULTS

### Stool samples

A total of 646 stool samples were collected from community-dwelling individuals across multiple regions in Japan between August and September 2023. Of these, 250 samples were obtained from the Chubu region, primarily Aichi Prefecture (*n* = 217), and 242 were collected from the Kanto region. Bacteria suspected to belong to the order *Enterobacterales* were detected in 627 of the 646 samples (97.1%), yielding a total of 1,340 isolates. Nineteen samples that did not grow any *Enterobacterales* were considered inadequate and excluded. The remaining 627 samples were used as adequate specimens for subsequent analyses.

### Species identification by ANI

Among the 1340 bacterial isolates, 407 strains detected from 368 samples (58.7%) were identified as *Klebsiella* species (Table S1). The species distribution was as follows: *K. pneumoniae* (*n* = 218, 53.6%), *K. variicola* (*n* = 137, 33.7%), *K. quasipneumoniae* (*n* = 51, 12.5%), and *K. quasivariicola* (*n* = 1, 0.25%). Of these, 39 samples contained two distinct *Klebsiella* species; 13 samples contained *K. pneumoniae* and *K. quasipneumoniae*; 25 samples contained *K. pneumoniae* and *K. variicola*; and one sample contained both *K. quasipneumoniae* and *K. quasivariicola* (Table 1). The estimated distribution of *Klebsiella* species based on random sampling was consistent with these findings. Among the 100 randomly selected specimens, *K. pneumoniae* complex species were detected in 60 samples, with the following species distribution: *K. pneumoniae* (n = 36, 60.0%), *K. variicola* (19, 31.7%), and *K. quasipneumoniae* (5, 8.3%) (Table 1; Table S2).

**TABLE 1:** Number of samples from which *Klebsiella* strains were isolated

Species detected	Number of samples (%)	Adjusted proportion (%)
*K. pneumoniae*	180 (48.9)	36
*K. quasipneumoniae*	37 (10.1)	5
*K. variicola*	112 (30.4)	19
*K. pneumoniae + K. quasipneumoniae*	13 (3.5)	NA^*a*^
*K. pneumoniae + K. variicola*	25 (6.8)	NA
*K. quasipneumoniae + K. quasivariicola*	1 (0.27)	NA
Total	368	60

^*a*^ NA, not applicable.

### Sequence types of *Klebsiella* spp.

**Fig 1:**
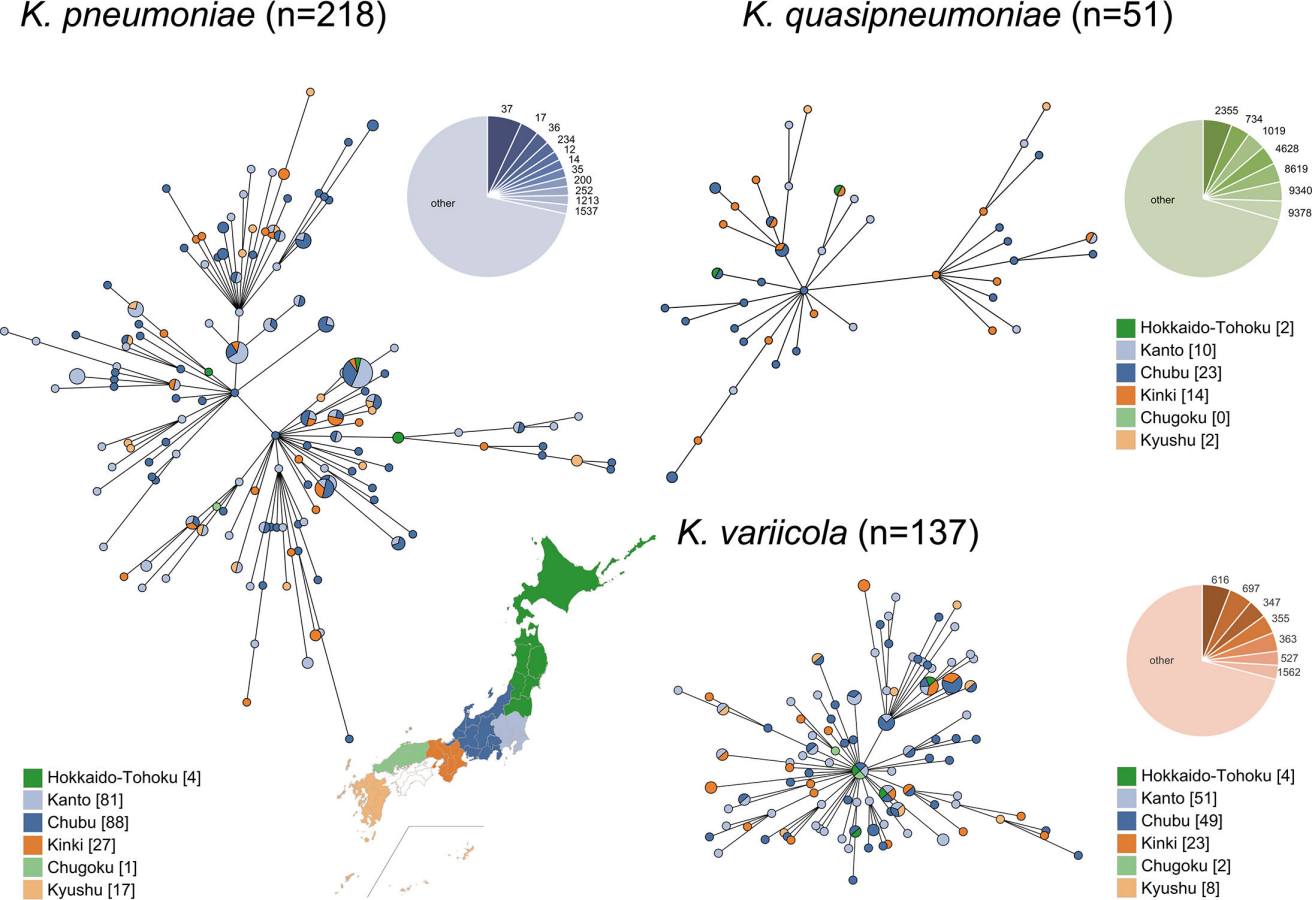
Geographical distribution of *Klebsiella* strains. Minimum-spanning trees generated using GrapeTree for each *Klebsiella* species based on MLST results. Circle colors represent the geographic regions where the subjects reside. Regional color coding in the trees corresponds to the map of Japan shown in the figure. Frequency of the sequence type of *K. pneumoniae, K. quasipneumoniae*, and *K. variicola* is also shown in the pie charts. The base map was obtained from https://www.kabipan.com/geography/whitemap/ distributed under a Creative Commons license, with source data derived from the Geospatial Information Authority of Japan.

**Fig 2:**
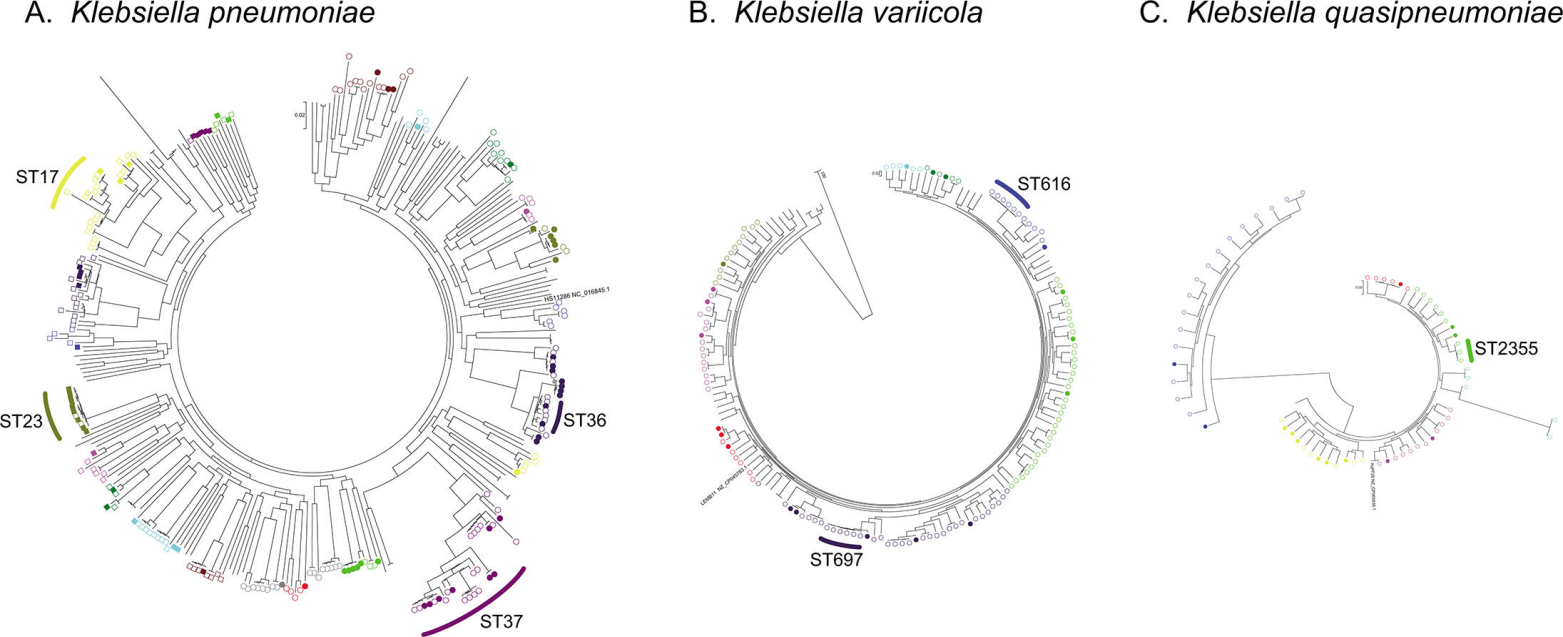
SNP-based phylogenetic trees incorporating strains isolated from bloodstream infections (BSIs). (**A**) *Klebsiella pneumoniae*, (**B**) *Klebsiella variicola*, and (**C**) *Klebsiella quasipneumoniae*. Clusters containing five or more strains are indicated by circles or squares. Same colors indicate clusters defined by TreeCluster. Strains identified in this study are shown with open symbols, while strains collected from BSI patients are indicated by filled symbols.

A total of 407 *Klebsiella* strains were classified into 285 distinct sequence types (STs). Among the 218 *K*. *pneumoniae* strains, ST37 was the most frequently identified (*n* = 15, 6.9%), followed by ST17 (*n* = 8, 3.7%) and ST36 (*n* = 6, 2.8%). Among the 137 *K*. *variicola* strains, ST616 was the most prevalent (*n* = 6, 4.4%), followed by ST697 (*n* = 5, 3.6%). In contrast, the 51 *K*. *quasipneumoniae* strains exhibited greater diversity. ST2355 was the most common ST within this species (*n* = 3, 5.9%), but 70.6% of strains belonged to distinct STs (Fig. 1). Overall, the majority of strains (54.8%) belonged to unique STs. Owing to the high degree of ST diversity and the low number of strains per ST across geographic regions, statistical comparison of ST distributions using the *χ*^2^ test lacked sufficient power and was, therefore, not considered appropriate for evaluating regional differences. Even so, no notable geographic clustering of STs was observed (Fig. 1). In the single nucleotide polymorphism (SNP)-based phylogenetic trees (Fig. 2), the frequencies of bloodstream infection (BSI)-derived strains differed significantly from those of stool-derived strains among clusters of *K. pneumoniae* (*P* = 0.00019). In contrast, no significant differences in the frequencies of BSI-derived strains among clusters were observed in the *K. quasipneumoniae* and *K. variicola* trees.

### Virulence genes and capsular genotypes

The capsular polysaccharide regulatory genes *rmpA* and *rmpA*2 genes were detected in three of 218 *K*. *pneumoniae* strains (1.4%). Two of these strains (FUJA0307 and FUJA0714) belonged to ST23 and the capsular genotype K1, and another (FUJA1256) belonged to ST412 and K57. All three strains also harbored the multiple iron acquisition genes, including *iro*BCDN (salmochelin siderophore biosynthesis), *iuc*ABCD (aerobactin siderophore biosynthesis), *iut*A (aerobactin transporter), and *peg-344* (metabolite transporter). Comparative analysis of hybrid genome assemblies from these three strains against the reference virulence plasmid pLVPK (GenBank Accession number AY378100) revealed the presence of plasmids closely related to pLVPK (Fig. S1). The plasmids from the two ST23 strains (FUJA0307 and FUJA0714) covered approximately 96% of pLVPK, while the plasmid from the ST412 strain (FUJA1256) covered about 84%. All virulence genes mentioned above were located on these plasmids in each strain. None of the strains of *K. variicola* and *K. quasipneumoniae* carried these virulence-associated genes.

### Resistance genes

Among the 407 strains, three strains (0.74%; FUJA1146, FUJA0986, and FUJA0800) harboring CTX-M type ESBL genes (*bla*_CTX-M-14_, *n* = 2 [ST37 and ST323] and *bla*_CTX-M-15_, *n* = 1 [ST9372]) were identified from isolates grown on MacConkey agar supplemented with cefotaxime. Antimicrobial susceptibility testing revealed that all three strains were resistant to cefotaxime and cefazolin, and the strain harboring the *bla*_CTX-M-15_ gene was also resistant to ceftazidime (Table 2). Furthermore, AMRFinderPlus detected *bla*_SHV-38_ as a potential ESBL gene in three *K. pneumoniae* strains (FUJA0024, FUJA1218, and FUJA1221). However, the strain did not exhibit an ESBL phenotype.

**TABLE 2:** Minimum inhibitory concentration among strains in which cephalosporin resistance genes were detected by AMRFinderPlus^*a*^^,*b*^

Strain no.	ST	β-Lactamase gene	MIC (μg/mL) of:										
			AMC	TZP	CEZ	CMZ	FMOX	CTX	CAZ	FEP	C/T	GEN	LVX
FUJA0800	9372	*bla*_CTX-M-15_	4/2	4/4	>8	≤0.5	≤0.12	16	16	32	0.5/4	≤2	≤0.5
FUJA0986	323	*bla*_CTX-M-14_	8/4	4/4	>8	1	≤0.12	8	≤1	2	0.5/4	>8	≤0.5
FUJA1146	37	*bla*_CTX-M-14_	16/8	4/4	>8	2	≤0.12	32	2	32	1/4	≤2	≤0.5
FUJA0024	9338	*bla*_SHV-38_	≤2/1	16/4	≤2	8	≤0.12	≤1	≤1	≤1	1/4	≤2	≤0.5
FUJA1218	827	*bla*_SHV-38_	≤2/1	4/4	4	1	≤0.12	≤1	≤1	≤1	0.5/4	≤2	≤0.5
FUJA1221	827	*bla*_SHV-38_	≤2/1	4/4	≤2	≤0.5	≤0.12	≤1	≤1	≤1	0.5/4	≤2	≤0.5

^*a*^ All strains are *K. pneumoniae.*

^*b*^ AMC, amoxicillin-clavulanate; TZP, piperacillin-tazobactam; CEZ, cefazolin; CMZ, cefmetazole; FMOX, flomoxef; CTX, cefotaxime; CAZ, ceftazidime; FEP, cefepime; C/T, ceftolozane-tazobactam; GEN, gentamicin; and LVX, levofloxacin.

## DISCUSSION

In this study, we investigated the distribution of *K. pneumoniae* complex species among individuals who resided in the community and were presumed to be healthy. Members of the *K. pneumoniae* complex were detected in approximately 60% of stool samples. This colonization rate is comparable to those reported in pregnant women in Cambodia (66.4%), where detection was based on rectal swabs or stool samples enriched in amoxicillin-supplemented medium, and in healthy individuals without recent healthcare exposure in several Asian countries (41.3% to 87.7%) (17, 18). In contrast, considerably lower colonization rates have been reported in Europe and North America. For example, a study from Norway documented a prevalence of 16.3% based on stool samples stored at −80°C from randomly selected citizens (19), and a U.S. study reported a rate of 23% among hospitalized patients in intensive care or hematology/oncology units (16). Although differences in culture media, colony selection strategies, and identification methods may influence detection rates, the findings of our study support the observation that colonization with *K. pneumoniae* complex species is generally more common in Asian populations. Lin et al. reported a colonization rate of 18.8% in Japan; however, that study included only 32 specimens collected from Chinese residents in Japan, which may not accurately reflect the general Japanese population. Our results align with previous reports suggesting that *K. pneumoniae* complex members may constitute part of the normal intestinal microbiota in these regions.

In this study, approximately half of the strains were identified as *K. pneumoniae* and one-third as *K. variicola*. Compared to previous studies analyzing clinical isolates, detection rates of *K. variicola* in our cohort were notably higher, as prior reports have documented rates ranging from 17.1 to 24.4% (10, 20, 21). Our findings suggest that *K. variicola* may constitute a component of the normal gut microbiota in the community. Certain *K. variicola* strains have been implicated in severe infections associated with high mortality rates (21, 22). However, no virulence genes were detected in any of the *K. variicola* strains in our study. Moreover, the genetic characteristics of the *K. variicola* strains isolated from BSIs in a previous study from Japan (10) were indistinguishable from those identified here. This indicates that the virulence potential of the *K. variicola* strains in our study is comparable to that of clinical isolates. A recent study suggested that *K. variicola* may generally exhibit lower virulence (23). Together, these findings support the notion that most *K. variicola* lineages detected in our study likely represent commensal strains within the intestinal microbiota, and that high-risk lineages appear to be rare in the community setting.

The most common STs of *K. pneumoniae* identified in this study were ST37 (6.9%) and ST17 (3.7%), consistent with patterns previously reported in clinical isolates (24–27). Several additional STs detected in this study have also been documented in clinical settings, suggesting that the overall ST distribution among individuals in the community may reflect that observed in clinical populations (25, 28–31). Although *Klebsiella* populations are generally characterized by high ST diversity without clearly dominant clones, ST37 is notable because it has been frequently reported in BSIs and is often associated with ESBL production (10, 32, 33). Thus, ST37 may represent an important clinical lineage with respect to both antimicrobial resistance and virulence. In contrast, some clusters in the SNP-based phylogenetic tree exhibited a lower frequency of BSI-derived strains. This observation suggests that commensal *Klebsiella* strains may include lineages with lower virulence potential compared to those commonly found in clinical isolates.

Notably, three hypervirulent *K. pneumoniae* strains (0.74%) were identified in this study, belonging to ST23-K1 and ST412-K57. These strains harbored virulence plasmids closely related to pLVPK, a well-known marker of hypervirulence. Despite their identification, the overall prevalence of hypervirulent lineages among community-dwelling individuals was substantially lower than that observed in clinical *K. pneumoniae* isolates based on MALDI-TOF MS identification, where approximately 20% were reported to carry *rmpA* (34). In contrast, a study from Korea reported a hypervirulent *K. pneumoniae* colonization rate of 4.6% among healthy individuals (35). Our findings suggest that colonization with hypervirulent *K. pneumoniae* strains is relatively uncommon in the community in Japan. Nevertheless, individuals colonized with hypervirulent *K. pneumoniae* strains may still be at increased risk of developing invasive infections compared to those harboring less virulent *Klebsiella* strains.

In recent years, the prevalence of ESBL-producing *K. pneumoniae* has been increasing among clinical isolates (36, 37). In contrast, ESBL-producing *K. pneumoniae* strains were rarely detected among individuals in the general community in this study, suggesting that ESBL-producing *K. pneumoniae* has not spread in the community in Japan. This is in contrast to *Escherichia coli*, for which significant carriage rates of ESBL-producing strains have been reported among healthy individuals in many countries including Japan (38, 39).

We acknowledge limitations of our study. Stool samples used in this study were obtained from a commercial clinical laboratory that routinely tests specimens submitted by clients, most of whom are food industry workers. However, no detailed information was available regarding the individuals who provided the samples, aside from the locations to which the test results were returned; therefore, duplicate sampling cannot be fully excluded. Although most samples were likely collected from healthy individuals, some may have originated from persons with underlying medical conditions, including those who had been recently hospitalized or were receiving antimicrobial therapy. In addition, the geographic distribution of the samples was skewed toward central Japan, which may have introduced a location-based sampling bias. Finally, only one representative isolate was further worked up from each stool sample unless different morphologies were observed, potentially underestimating the genetic diversity of *K. pneumoniae* complex in these stool samples.

In conclusion, *K. pneumoniae* complex strains colonized approximately 60% of the community-dwelling individuals in Japan. The overall ST distribution was similar to that of clinical isolates, but hypervirulent and ESBL-producing strains were rare and each comprised less than 1%. The findings suggest that, although carriage of *K. pneumoniae* complex is common, transmission of high-risk *K. pneumoniae* strains is predominantly occurring in healthcare than community settings in Japan.

## MATERIALS AND METHODS

### Stool samples

Stool samples were collected from community-dwelling individuals across multiple regions in Japan. These samples were submitted to a commercial diagnostic laboratory mostly by workers in the food industry on a routine basis to rule out *Shigella* spp., *Salmonella* Typhi, *Salmonella* Paratyphi A, and enterohemorrhagic *E. coli*. Residual stool samples received over a 6-week period between August and September 2023 were transported to the research laboratory once a week for processing. The study was approved by the institutional review board of Fujita Health University on an opt-out consent basis (HM23-065).

### Screening using selective isolation media

Stool samples were plated onto MacConkey agar supplemented with 1 µg/mL cefotaxime, as well as deoxycholate hydrogen sulfide lactose (DHL) and *Salmonella*-*Shigella* (SS) agar plates. The plates were incubated at 37°C for 24 h. Up to five colonies displaying morphological characteristics consistent with *E. coli* or *Klebsiella* spp. were selected for further analysis. Selected colonies were subcultured onto CHROMagar Orientation plates and incubated under the same conditions. Colonies exhibiting metallic blue coloration were considered presumptive members of the *K. pneumoniae* complex; however, this phenotype may also include other *Enterobacterales*, such as *Enterobacter* spp. Stool samples from which any *Enterobacterales* were detected were considered valid for inclusion in the study.

### Identification of *Klebsiella* species by PCR

Presumptive *Klebsiella* isolates were subjected to species-level identification using polymerase chain reaction (PCR) with *Klebsiella*-specific marker primers: (*K. pneumoniae*, F: 5′-TGACTGCGTTGTAAAAAGCG-3′, R: 5′-AATTTAGGTTTACCGTCTGCG-3′, *K. variicola*, F: 5′- ATGCAGGCCAATTTCGAC-3′, R: 5′-CCATGGCCAAATCGACTT-3′, and *K. quasipneumoniae*, F: 5′-ACGGAACATTCTCTCTGAAGCC- 3′, R: 5′-ACAGATTTAAAGGCGCTGGA-3′) (40). Template DNA was prepared by suspending bacterial cells in Tris-EDTA buffer at McFarland 0.5, followed by heat lysis at 95°C for 10 min and centrifugation. The resulting supernatant was used as the PCR template. PCR reactions were performed using GoTaq Hot Start Polymerase (Promega) following the manufacturer’s protocol. Thermal cycling conditions were 94°C for 30 s, 60°C for 30 s, and 72°C for 1 min, repeated for 30 cycles. Amplicons were visualized via electrophoresis on 2.5% (w/v) agarose gels stained with ethidium bromide and examined under UV illumination.

### Whole genome analysis

Whole-genome sequencing was performed on PCR-confirmed *Klebsiella* strains. Duplicate strains of the same species from the same individual were excluded. Genomic DNA was extracted using the Gentra Puregene Yeast/Bact Kit (Qiagen). DNA libraries were prepared with the QIAseq FX DNA Library Kit (Qiagen) and sequenced on the Illumina NextSeq 2000 platform (Illumina, San Diego, CA, USA). Reads were assembled using SPAdes version 3.13.1. Strains carrying the *rmpA* gene additionally underwent long-read sequencing using Oxford Nanopore MinION (O. Hybrid assemblies were generated with Unicycler v0.5.1 using both Illumina and Nanopore reads, with the depth_filter parameter adjusted to 0.1 to prevent the loss of low-coverage sequences.

### Species identification

Species-level identification was carried out using FastANI (https://github.com/ParBLiSS/FastANI) (41). Strains showing average nucleotide identity (ANI) value of 95% or higher against a reference genome (*K. pneumoniae* strain HS11286 [CP003200], *K. quasipneumoniae* strain KqPF26 [CP065838], *K. variicola* strain LEMB11 [CP045783], and *Klebsiella quasivariicola* strain KPN1705 [CP022823]) were classified as the same species (42).

### Rate of *K. pneumoniae* complex species using randomized samples

The number of colonies selected from each sample varied depending on the culture conditions. To estimate the prevalence of *K. pneumoniae* complex species, 100 specimens were randomly selected, and the number of *Klebsiella* isolates recovered from each sample was recorded. For samples containing multiple *Klebsiella* species, a single strain was randomly chosen for subsequent analyses.

### Genotypic characterization

Multilocus sequence typing (MLST) was performed in accordance with protocols from the Institut Pasteur MLST and Whole-Genome MLST Database (https://bigsdb.pasteur.fr/klebsiella/). Geographical distribution was visualized using minimum-spanning trees based on MLST profiles generated with GrapeTree (https://github.com/achtman-lab/GrapeTree). To assess regional differences in ST distribution, a *χ*^2^ test was conducted comparing the Chubu and Kanto areas using STs represented by three or more isolates. Capsular genotypes were determined based on *wzi* sequences using the kaptive command-line interface (https://github.com/klebgenomics/Kaptive). Antimicrobial resistance genes, including those conferring cephalosporin resistance, as well as virulence genes, were detected using AMRFinderPlus with the National Center for Biotechnology Information Bacterial Antimicrobial Resistance Reference Gene Database (43). Kleborate 3.2.4 (https://github.com/klebgenomics/Kleborate) was also used to determine additional genetic features. Core genome single nucleotide polymorphism (SNP) analysis was performed using SNIPPY version 4.6.0, incorporating strains isolated from BSIs in our previous study (10). Phylogenetic trees for each species were constructed using FastTree version 2.2 and visualized with MEGA version 7.0.26. Clusters within the phylogenetic trees were defined using TreeCluster version 1.0.4 (https://github.com/niemasd/TreeCluster). The frequency of BSI-derived strains within clusters consisting of five or more strains was evaluated using the *χ*^2^ test.

### Antimicrobial susceptibility testing

Antimicrobial susceptibility testing was conducted using the broth microdilution method in accordance with the Clinical and Laboratory Standards Institute (CLSI) guidelines. Minimum inhibitory concentrations (MICs) were determined using *K. pneumoniae* ATCC 700603 as the quality control strain. The antimicrobial agents tested included: amoxicillin-clavulanate, piperacillin-tazobactam, cefazolin, cefmetazole, flomoxef, cefotaxime, ceftazidime, cefepime, ceftolozane-tazobactam, gentamicin, and levofloxacin.

## Data Availability

The genome sequencing data presented in this work are deposited under BioProject accession number PRJDB35784.

## SUPPLEMENTAL MATERIAL

Fig. S1 aem.01687-25-s0001.pdf Plasmid map.

Supplemental tables aem.01687-25-s0002.xlsx Tables S1 and S2.

ASM does not own the copyrights to Supplemental Material that may be linked to, or accessed through, an article. The authors have granted ASM a non-exclusive, world-wide license to publish the Supplemental Material files. Please contact the corresponding author directly for reuse.
