# Supplementary material for: Carriage of hypervirulent and ESBL-producing Klebsiella pneumoniae complex among community-dwelling individuals in Japan
Source: Appl Environ Microbiol. 2026 Jan 14;92(2):e01687-25. doi: 10.1128/aem.01687-25 (PMC12915344; doi:10.1128/aem.01687-25)
Supplement: Fig. S1 — Plasmid map. [file aem.01687-25-s0001.pdf]

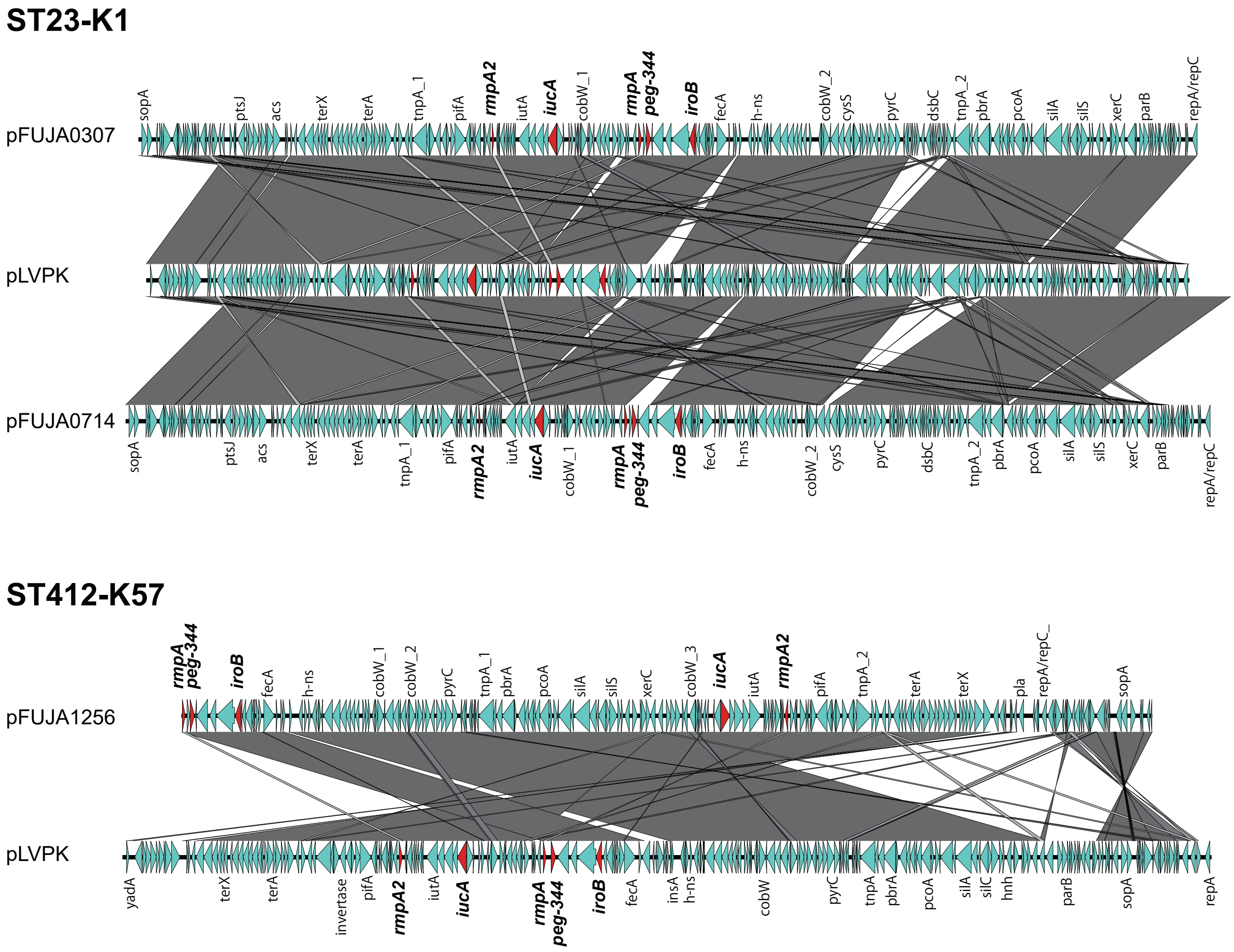

Figure S1. Comparison of virulence plasmids in ST23-K1 strains (FUJA0307 and FUJA0714) and the ST412-K57 strain (FUJA1256) with *Klebsiella pneumoniae* CG43 pLVPK.

Five reference virulence genes, *rmpA*, *iucA*, *peg344*, *rmpA2*, and *iroB*, are highlighted in red.
